# Diet After Acute Coronary Artery Syndrome

**DOI:** 10.3390/nu18010005

**Published:** 2025-12-19

**Authors:** Vasiliki Katsi, Marilena Giannoudi, Vasilios G. Kordalis, Konstantinos Tsioufis

**Affiliations:** 1 Cardiology Department, School of Medicine, Hippokration General Hospital, National and Kapodistrian University of Athens, 157 72 Athens, Greece; vkkatsi@yahoo.gr (V.K.);; 2 Leeds Institute for Cardiovascular and Metabolic Medicine, University of Leeds, Leeds LS2 9JT, UK; 3 School of Medicine, Aristotle University of Thessaloniki, 541 24 Thessaloniki, Greece

**Keywords:** acute coronary syndrome, diet, secondary prevention, Mediterranean diet, dietary modification, cardiovascular disease, lifestyle intervention

## Abstract

**Background:** Acute coronary syndrome (ACS) encompasses ST-elevation myocardial infarction, non-ST-elevation myocardial infarction, and unstable angina. While optimal medical therapy (OMT) is central to secondary prevention, lifestyle interventions—particularly dietary modification—remain underutilised despite their potential impact on long-term outcomes. **Objective:** To review the current evidence regarding dietary interventions post-ACS, their implementation, adherence, and effects on cardiovascular risk factors and clinical outcomes. **Methods:** A narrative literature review was performed using PubMed, including studies published in English from 2000 onwards. Keywords included “acute coronary syndrome,” “diet,” “cardiovascular disease,” “outcomes,” “adherence,” “wine,” and “intermittent fasting,” combined with Boolean operators AND/OR. Animal studies were excluded. The latest search was conducted in October 2025. **Results:** Mediterranean-style diets, when combined with OMT and lifestyle interventions (exercise, smoking cessation, alcohol moderation), consistently improve cardiovascular risk factors and reduce recurrent ischemic events and mortality. Clinical trials and cohort studies demonstrate long-term benefits, including reductions in all-cause mortality and major adverse cardiovascular events, particularly in patients adhering to structured dietary programmes within cardiac rehabilitation. Evidence for other dietary modifications, including low-fat diets, increased fibre, antioxidant supplementation, and intermittent fasting, was more limited, often derived from small or short-term studies focusing on surrogate endpoints. Real-world adherence to dietary guidelines remains suboptimal, especially in high-risk and obese populations. Preliminary studies suggest intermittent fasting and moderate red wine consumption may confer additional cardiovascular benefits, though larger, long-term trials are needed. **Conclusions:** Dietary modification is a key, yet underutilised component of secondary prevention post-ACS. A Mediterranean-style, whole-food diet integrated with OMT and supported by structured cardiac rehabilitation programmes offers the most evidence-based strategy to improve risk factor control and long-term outcomes. Future research should focus on pragmatic, long-term trials assessing hard cardiovascular endpoints and implementation strategies to enhance adherence across diverse populations.

## 1. Introduction

Acute coronary artery syndrome (ACS) comprises ST-elevation myocardial infarction (MI), non-ST-elevation MI (NSTEMI), and unstable angina. After a diagnosis such as this, importance must be placed on establishing secondary prevention measures for the long-term management of these patients. Although substantial emphasis has been placed on the medical management of patients following ACS, lifestyle interventions, including dietary modification, remain a critical and often underappreciated component of secondary prevention. This review will outline the latest research, guidelines, and updates regarding diet post-ACS. This is of particular importance as a recent systematic analysis conducted in a Global Burden of Disease (GBD) study revealed cardiovascular disease (CVD) as the primary cause of mortality and disability-adjusted life-years attributed to diet [1].

Dietary intake is fundamental to both the provision of essential nutrients for optimal health and the control of modifiable cardiovascular risk factors in patients with cardiac disease. Eating choices and habits have been linked to blood pressure, cholesterol, and glycaemic control, in addition to weight regulation. In particular, energy intake limited to the amount needed to obtain and maintain a healthy weight (BMI < 25 kg/m^2^), and increasing physical activity, are recommended for weight management after ACS for improved long-term outcomes [2]. The aforementioned variables are key risk factors for the development of primary cardiac events and should be optimised for effective primary and secondary prevention.

## 2. Current Secondary Prevention Guidelines

According to the European Society of Cardiology (ESC) guidelines, secondary prevention after an ACS event should be offered to every patient, as early as possible after their index event. Information leaflets aimed at patients and endorsed by the society in conjunction with Oxford University Press state that patients should ‘Eat Healthy. Try to eat a balanced Mediterranean-type diet, with lots of fruit and vegetables’ [3]. In keeping with this, the current international dietary recommendations, such as the Dietary Approaches to Stop Hypertension (DASH) [4] and the American Heart Association (AHA) guidelines [5], also recommend a Mediterranean-style diet, describing the need for a holistic change in the dietary pattern. This should include fruits, vegetables, nuts, legumes, fish, vegetable oil, low-fat dairy, and whole grains, and limit red meats, processed meats, refined grains, added sugars, and salt. Finally, the ESC suggests that dietary changes should be accompanied by alcohol restriction of up to 100 mL/week, irrespective of biological sex. Implementing these proposed changes alongside other lifestyle behaviours, such as regular exercise and tobacco abstinence, decreases the risk of subsequent cardiovascular events and mortality and contributes to holistic secondary prevention therapy [3].

Table 1 summarises the principal dietary patterns and the proposed cardiovascular benefits associated with their constituent food groups. Over the past two decades, there has been a proliferation of celebrity-endorsed and eponymous diets; however, these fall outside the scope of this review.

Unfortunately, compliance with the guidelines remains poor in many countries, particularly in populations with higher cardiovascular disease risk [38,39,40]. A large cross-sectional study involving 78 centres across 24 European countries, which interviewed patients at the time of their index ACS event, after coronary artery bypass grafting (CABG) or percutaneous coronary intervention (PCI) treatment for coronary artery disease in the absence of an ACS event, and again at 6 months post-event, revealed that the majority of these patients did not achieve the guideline standards for secondary prevention. A substantial proportion of patients continued to engage in unhealthy behaviours, with 48.6% reporting persistent smoking, 59.9% physical inactivity, and widespread unhealthy dietary patterns. Consequently, a high proportion remained overweight or obese (37.6%), alongside a notable prevalence of diabetes (26.8%) [41]. Furthermore, there was a large variation in the secondary prevention practice between the different centres. Risk factor control remained inadequate despite the high reported use of the necessary secondary prevention medications (93.8% antiplatelets, 82.6% betablockers, 75.1% angiotensin converting enzyme inhibitors/angiotensin receptor blockers, and 85.7% statins) and lifestyle interventions, particularly in the obese cohorts. The majority of patients had attempted to change their diet since their coronary event by reducing the intake of salt (71.8%), fat (78.9%), sugar (66.1%), alcohol (53.5%) and calories (63.3%), in addition to changing fat composition (71.1%) and increasing fruits and vegetables (77.8%) and fish (66.8%) consumption. Unfortunately, this pattern was not followed by the obese patient group, in which less than half (48.1%) of patients had followed dietary recommendations since their coronary event, 48.4% increased their regular physical activity levels to lose weight, and 3.0% used appetite suppressants or other anti-obesity medications. Only half of the participants (49.8%) tried to lose weight within the month prior to the interview, and less than two-thirds (61.9%) reported considering losing weight within the next six months. However, approximately one in five of the obese patients reported never being told they were overweight. This evidence indicates a demand for more practical strategies in nutritional care regarding major food sources that contribute most to cardiovascular risk in a particular population of interest and highlights the need for comprehensive secondary prevention care across populations [41]. Although the findings provide valuable insights, they should be interpreted with caution, given the cross-sectional design and reliance on self-reported lifestyle data, which are susceptible to recall and social desirability bias. Notably, as the majority of participants reported suboptimal behaviours and outcomes, it is plausible that the true extent of lifestyle non-adherence and risk factor burden may be underestimated in the published results.

In this review, we set out to investigate the current evidence in the field surrounding the guideline recommendations and assess whether it is adequate to explain how diet can affect outcomes within the post-ACS patient setting.

## 3. Methods

We performed a narrative literature review to explore how dietary changes are implemented, adhered to and affect prognosis post-ACS events. The search was conducted on PubMed, using the following keywords: acute coronary syndrome, diet, cardiovascular disease, outcomes, adherence, wine, as well as intermittent fasting, whilst simultaneously utilising the Boolean operators AND and OR. All article types written in English from 2000 onwards were considered. Any research on animals was excluded. The latest search took place in October 2025.

## 4. Diet for Secondary Prevention of Ischaemic Cardiac Events and Prognosis

When considering the dietary aspect of secondary prevention, a systematic review by Skinner et al. presented evidence relating to the effectiveness and safety of numerous dietary interventions, including eating less fat, more fibre and fish oils, as well as the consumption of a Mediterranean diet and vitamins, increased exercise output, and ensuring optimal medical therapy (OMT) was prescribed [42]. The authors highlight that the magnitude of cardiovascular risk reduction in people with coronary artery disease correlated directly with the magnitude of blood pressure reduction and was achieved through appropriate up-titration of anti-hypertensive medication. However, with regard to lifestyle changes, they stated that ‘cardiac rehabilitation (including exercise) and smoking cessation reduce the risk of cardiac events in people with CHD [coronary heart disease]’ [42].

Such a drive towards the integration of lifestyle changes in the field of cardiovascular medicine has led to the introduction of a new field of cardiology called behavioural cardiology [43,44]. This is intended to emphasise the role of psychosocial, behavioural, and lifestyle factors in shaping cardiovascular health outcomes. As such, there is a drive for clinicians to routinely assess lifestyle behaviours (diet, activity, sleep, substance use) as “vital signs” in clinical encounters. Rozanski et al. highlighted the importance of early “windows of opportunity” when patients first encounter health services (including post-ACS events) to embed behavioural evaluations. They also promoted the expansion of cardiac rehabilitation into more accessible, lower-cost formats, and extended eligibility to those with cardiovascular risk factors (even in the absence of established disease) [44].

Evidence from clinical trials indicated that, in patients following acute coronary syndrome, adherence to a Mediterranean diet compared with a Western dietary pattern was associated with a reduced risk of all-cause mortality and the composite endpoint of cardiac death and non-fatal MI. Importantly, these data originated from a randomised, controlled trial with a follow-up duration of 46 months, highlighting the sustained long-term benefits of this dietary modification. There remains inadequate evidence for the advice of increasing dietary fibre intake and reducing dietary fat intake based on the published data, with no statistically significant results in improving mortality or further myocardial infarction events in patients who adhered to these changes compared to those who did not. Furthermore, antioxidant vitamins (including vitamin E, beta-carotene, or vitamin C) had no effect on cardiovascular events in patients with prior ACS, compared with placebo [42].

In a large cohort study performed in Bordeaux, France, investigators recruited just under 1000 post-ACS patients and subjected them to OMT in combination with an exercise and dietary educational programme. Participants were advised to restrict total fat intake to <30% of daily energy intake, with saturated fatty acids contributing < 10%. A dietician provided patient-specific weight management guidance. Educational classes were given with regard to all aspects of high-risk coronary artery disease patients. Light-to-moderate exercise for at least 30 min three times weekly was recommended. Cardiovascular risk factors (body weight, blood pressure, and lipid and glycaemic indices) and markers of atherosclerotic disease, including intima–media thickness, carotid atheroma, and peripheral arterial disease, were evaluated at three months post-acute coronary syndrome, with cardiovascular events monitored over a total follow-up duration of 20 months. At follow-up, using the combination of OMT alongside dietary and exercise interventions, >80% of participants had reached their secondary prevention goals. Notably, direct adherence to OMT was not assessed, but rather, the team reviewed the participants’ repeat prescriptions to review medication compliance. Nonetheless, within this population, the presence of diabetes was the only cardiovascular risk factor significantly associated with CV events in multivariate analysis, including traditional risk factors (hazard ratio [HR] 1.61, *p*  =  0.017). However, in multivariate analyses, which included all the initially measured cardiovascular risk factors and atheroma disease markers, only peripheral artery disease remained significantly associated with cardiovascular events (HR 1.83, *p* = 0.04). Unsurprisingly, the higher the number of vascular beds involved, the worse the overall prognosis of the patient. This study highlighted the importance of combined medical and lifestyle intervention for long-term prognosis and improved morbidity and mortality in the post-ACS cohort [45].

A similar multicentre study by Rodríguez et al. was conducted to investigate whether adherence to OMT was sufficient, or if the addition of a healthy lifestyle provided additional clinical improvement for 685 patients post-ACS [46]. The participants were given standard healthy lifestyle advice following ESC guidelines [3] on secondary prevention. This recommended their adherence to the following variables: intake of ≥3 fruits and vegetables/day, ≥2 fish servings/week, ≤7 alcohol beverages/week, feeling stress < once/month, moderate–intense physical activity in leisure time, walking at work, and giving up tobacco. Each of these variables was measured as per the ‘SCORE’ criteria (each variable was granted a score of 1). Over a short mean follow-up time of 4.89 years, a lifestyle SCORE ≥ 4 was independently and inversely associated with both the incidence of the primary outcome (ischemic events [any ACS, stroke, or Transient Ischemic Attack] or death) (HR 0.65 (95% confidence interval [95% CI] 0.44–0.96); *p* = 0.029) and death (HR 0.41 [95% CI 0.18–0.91]; *p* = 0.029). Furthermore, Kaplan–Meier curves showed a higher event-free survival for the aforementioned outcomes in patients with a SCORE ≥ 4 (healthy lifestyle) than in those with a SCORE < 4 (unhealthy lifestyle), although the short follow-up time of less than 5 years may have limited mortality generalisability. In this single-centre observation study, patients with a SCORE ≥ 4 had the additional benefit of a significantly greater decrease in total cholesterol [46].

Overall, the evidence supports a synergistic strategy combining OMT with diet and lifestyle changes. A prior non-randomised observational study was designed to evaluate the impact of a cardiovascular rehabilitation programme, including dietary counselling regarding adherence to dietary recommendations. The cohort was divided into two groups: Group I, comprising patients in the acute phase of ACS, and Group II, comprising patients six months to three years following the completion of a cardiovascular rehabilitation programme. Patients’ dietary intake was investigated using a food frequency questionnaire, which included scores for the consumption of saturated fatty acids, monounsaturated fatty acids, Omega-6 and Omega-3 polyunsaturated fatty acids, fruits and vegetables, and a global cardiovascular protective dietary score, as well as biological markers. The consumption of saturated fatty acids was higher in Group I vs. II, whereas Omega-3 polyunsaturated fatty acids, fruits and vegetables score, and global dietary score were higher in Group II. Furthermore, biological markers showed higher plasma contents of folate and vitamin C. This study, therefore, highlights that there can be sustained improvement in dietary habits in patients with coronary artery disease who receive nutritional education during a cardiovascular rehabilitation programme [47].

## 5. The Importance of the Mediterranean Diet

Whilst not directly assessing cardiac outcomes, Thomazella et al. have conducted a randomised control trial, comparing the Mediterranean diet to the low-fat Therapeutic Lifestyle Changes Diet alongside a dietician’s advice in post-ACS patients [48]. The aim of the study was to investigate the effects of the two diets on biochemical markers of cardiac health, including endothelial function, oxidative stress, and inflammation after ACS [48].

A comparison was conducted between a three-month Mediterranean diet and a low-fat Therapeutic Lifestyle Changes (TLC) diet in a cohort of male post-ACS patients, with each diet administered under a strategy designed to optimise adherence, which was documented at >90%. Interestingly, both diets promoted similar decreases in body mass index and blood pressure (*p* ≤ 0.001). The two diets did not further enhance flow-mediated brachial artery dilation compared to baseline. The Mediterranean diet decreased low-density lipoprotein and oxidised low-density lipoprotein plasma levels, although the ratio of oxidised to total low-density lipoprotein remained unaltered. Furthermore, this diet promoted an increase in high-density lipoprotein levels (*p* = 0.053). Glucose, high-sensitivity C-reactive protein, triglycerides, myeloperoxidase, intercellular adhesion molecular, vascular cell adhesion molecule, and glutathione serum and plasma levels remained unchanged with either diet. The presented evidence, therefore, suggests that balanced, low-fat diets after ACS can improve markers of redox homeostasis and metabolic effects potentially related to atheroprotection [48]. Further work in the field, across different post-ACS patient cohorts assessing such biomarkers, can help to identify which patients would benefit the most from such a dietary intervention.

Furthermore, one of the main complications associated with ACS is heart failure (HF). Kouvari et al. investigated the effect of the implementation of a Mediterranean diet in post-ACS patients with subsequent HF, which were classified, as per ESC criteria, as the following: reduced [HFrEF], mid-range HF [mrEF], and preserved ejection fraction [HFpEF] [49]. A cohort of 1000 consecutive patients admitted with a diagnosis of ACS was studied, with longitudinal follow-up spanning 10 years. A nutritionist was responsible for the assessment of dietary habits through the use of a semiquantitative food frequency questionnaire, which consisted of 75 items. Adherence to the Mediterranean diet was assessed through the validated MedDietScore, ranging between 0 and 55, with higher scores being associated with better adherence. Multivariate logistic regression analysis demonstrated that higher adherence to the Mediterranean diet was associated with a reduced incidence of fatal and non-fatal ACS events at 1, 2, and 10 years. In patients with HFrEF, a high MedDietScore was protective against recurrent cardiac episodes in the first 2 years post-index event, but this was no longer the case when the follow-up increased to 10 years. Whilst the results may once again be subject to recall or social desirability bias, given the self-reported nature of data collection, the authors concluded, however, that a higher MedDietScore was protective against recurrent cardiac episodes in the long-term prognosis of patients with HFmrEF and HFpEF [49].

## 6. Intermittent Fasting

Any comprehensive review of dietary interventions would be incomplete without the consideration of intermittent fasting. Although not a diet in the traditional sense of restricting food types, it imposes temporal limitations on food consumption throughout the day. Intermittent fasting is significant in that it may be adopted for health promotion and weight management, as well as for lifestyle or cultural reasons. For example, skipping breakfast, working shifts, or fasting during Ramadan.

The definition of intermittent fasting can encompass various categories, including alternate-day or whole-day fasting, as well as time-restricted feeding [50]. The aforementioned fasting regimes typically consist of no dietary intake at the time of intended fast, whereas time-restricted feeding consists of a fasting and a feeding window with the same eating routine each day. Multiple cardiovascular health benefits have been shown with this method, including a reduction in total fat mass and fat-to-lean ratio [51,52]. These anthropometric differences, alongside total body-weight reduction, have been shown across the full spectrum of baseline weight characteristics (i.e., in obese, overweight, and normal weight individuals). There is also evidence to suggest an improvement in cholesterol profiles, with a reduction in total cholesterol, triglycerides, and total low-density cholesterol also being observed [51].

Of relevance to the current review, intermittent fasting has been studied in the post-ACS context. Dutzmann et al. performed a randomised control trial investigating the effects of intermittent fasting on left ventricular ejection fraction as a marker of cardiac function. During a total follow-up time of 6 months, patients were randomised to adhere to intermittent fasting (comprising a regular diet for up to 8 h per day whilst fasting for the remaining 16 h for 3 months from baseline), or to a regular diet. The improvement in ejection fraction showed an ongoing incremental improvement over the course of 6 months. Furthermore, the intermittent fasting group showed a greater reduction in diastolic blood pressure and body weight. Importantly, the intermittent fasting group admitted to a high level of adherence to this diet (median 83.7%) [53]. Such results are promising for this method of dietary intake post-ACS, but more long-term follow-up and mechanistic research are required before considering this guideline-recommended therapy.

## 7. Alcohol Consumption

Consideration of alcohol consumption is imperative when reviewing dietary habits and the cardiovascular system. Published evidence suggests that the consumption of red wine can be beneficial, both for primary and secondary prevention of cardiovascular disease. In the post-ACS setting, Rifler et al. conducted a randomised study in which patients were divided into ‘red wine drinkers’ or ‘water drinkers’ during a three-week cardiac rehabilitation programme. This allowed the participants to experience the same diet (named the ‘Western prudent’, a Mediterranean-based regime) and exercise setup. The ‘red wine drinkers’ received two glasses of red wine per day (a total of 25 cl), with one glass being given at lunch and the other with the evening meal. Blood samples were collected at baseline and day 15 to assess lipid profiles, albumin, glucose, and oxidative status (through the evaluation of cytokine levels). At baseline, there were no differences in the values of the groups. However, by day 14, there was a significant decrease in total cholesterol in the ‘red wine drinkers’ compared to the ‘water drinker’ control group (−16%, *p* = 0.042). Furthermore, within the intervention group, there was a significant decrease in total cholesterol and LDL (*p* = 0.047 and *p* = 0.012, respectively) over the study period of intervention. The results of the antioxidant testing were less pronounced, and certainly, there were no anthropometric differences between baseline and day 14 of the two groups, as measured by body weight and systolic and diastolic blood pressures [54]. This work clearly highlights some beneficial effects of red wine consumption post-ACS, which cannot be overlooked given the strict control of diet and exercise between the two groups.

In a similar vein, Guarda et al. conducted endothelial function studies in patients post-ACS after being randomised to either drinking red wine or abstain from the beverage. In particular, they investigated flow-mediated vasodilatation of the brachial artery, plasma antioxidant capacity measured by total antioxidant reactivity, and ferric-reducing antioxidant power and oxidative damage, measured by 8-OH deoxyguanosine content in leukocyte deoxyribonucleic acid. The consumption of red wine led to a reduction in oxidative stress in the red wine drinking group, compared to the control group, from baseline to the two-month follow-up visit. However, there was no improvement in endothelial function in either group [55]. Whilst this study is not directly comparable to that of Rifler et al., given that they did not place guidance or restrictions on diet and exercise, and the participants were known to consume ‘moderate’ alcohol quantities prior to recruitment. Once again, however, the evidence suggests a potential benefit in wine consumption post-ACS. The results, however, must be considered with caution, given that they were only short-term, had no impact on anthropometric measurements, and had no known impact on clinical outcomes (short- or long-term). Furthermore, both studies consisted of small patient populations (fewer than 40 in both). There is therefore a need for larger studies, with longer-term follow-up, prior to the consideration of implementing alcohol advice in the post-ACS setting.

## 8. Diet and Primary Prevention of ACS

While the focus of this article is secondary ACS prevention, acknowledging primary prevention is essential for completeness. Primary prevention focuses on strict risk factor control to prevent cardiac events, and this is highlighted in both the European and American guidelines [56,57,58]. These include optimising blood pressure and blood glucose levels. A systematic review by Uthman et al. of 139 randomised control trials, representing over 1,000,000 participants, presented evidence that blood pressure treatments with intense targets, alongside lipid-lowering therapies including statins, are the most effective strategies for primary prevention [59]. Whilst there are some limitations to the work, including a high heterogeneity of the study populations in each trial, few head-to-head comparisons of the different interventions, and a search cut-off at 2021 (therefore missing newer treatments), the study continues to reflect the recommendations from the international guidelines.

Once again, the Mediterranean diet is considered imperative to reduce the 10-year risk of cardiovascular disease onset [60,61,62]. Certainly, guidelines set out by the American Diabetes Association and the American Heart Association support the notion of a balanced diet with the aim of controlling blood pressure and lipid levels [57]. Importantly, sodium restriction, combined with an increased consumption of fibre, fruit, vegetables, and calcium, has proven more effective than sodium restriction alone in reducing hypertension [63]. Furthermore, societies support minimal dietary fat intake, particularly of saturated fat, trans fats, or cholesterol, as this is associated with an increased risk for coronary artery disease and should be avoided [57,64]. Finally, Rojas et al. have also shown the importance of extra-virgin olive oil consumption in the prevention of cardiovascular disease (including myocardial infarction, stroke, peripheral arterial disease, heart failure, atrial fibrillation, or cardiovascular death) in over 7000 patients over a median follow-up of 4.7 years. In their observational study, participants with the highest annual consumption rates of extra virgin olive oil had a 25% lower risk of the composite outcome, i.e., any of the aforementioned outcomes (HR: 0.75; 95% CI: 0.60–0.94), with significant reductions in the individual cardiovascular disease outcomes [65].

## 9. Discussion

This review highlights the important role of dietary modification in the secondary prevention of ACS, whilst exposing several limitations in the existing evidence base. Although multiple dietary patterns have been evaluated—most notably Mediterranean, DASH, intermittent fasting, and low-carbohydrate strategies—the strength and quality of supporting studies vary considerably. Much of the evidence derives from small, short-term trials focused on surrogate biochemical endpoints, such as blood lipid profiles, oxidative stress markers, or endothelial function. While these provide useful mechanistic insight, they are insufficient to determine long-term clinical benefit. Only a limited number of larger studies demonstrate reductions in hard cardiovascular outcomes, and these are largely restricted to Mediterranean-style interventions.

The reliance on narrative review methods further underscores uncertainty. The searches were limited to one database, and the absence of a formal risk-of-bias assessment or predefined inclusion criteria introduced selection bias. Considerable heterogeneity existed across interventions, adherence strategies, comparators, and follow-up durations, which complicated direct comparison between dietary approaches. Additionally, many studies depended on self-reported dietary intake, which was prone to misclassification and confounding from broader lifestyle behaviours. Furthermore, real-world adherence remained suboptimal in large international samples, and important gaps persisted between guideline recommendations and patient behaviour—particularly in higher-risk and obese populations [12]. The evidence base for some specific dietary prescriptions (for example, isolated increases in fibre or blanket low-fat advice) was mixed, and antioxidant supplementation had not demonstrated benefit [13,17]. Heterogeneity in interventions, the variable duration of follow-up across studies, and differences in how “healthy” diets were implemented limit firm conclusions about the relative superiority of every dietary approach beyond the broader pattern of plant-forward, unprocessed foods and reduced processed/red meat intake.

Despite these limitations, the collective evidence consistently supported diets rich in vegetables, fruits, whole grains, legumes, and unsaturated fats. However, real-world adherence remained low, highlighting critical implementation barriers.

Future research should prioritise large, pragmatic trials with clearly defined interventions, objective adherence assessment, and long-term clinical endpoints. Equally important are implementation studies evaluating scalable, equitable strategies within cardiac rehabilitation and primary care. Overall, while dietary modification remains a central pillar of secondary prevention, the field requires more robust evidence to optimise its clinical application.

## 10. Conclusions

Dietary change is a crucial and under-utilised component of secondary prevention after ACS. The balance of evidence presented in this review indicates that adopting a Mediterranean-style dietary pattern, when combined with OMT and other healthy behaviours (exercise, smoking cessation, alcohol moderation), is associated with improvements in risk factors and with lower rates of recurrent ischaemic events and mortality in many cohorts and randomised studies [3,7,13,16,17]. Cardiac rehabilitation programmes led by a multi-disciplinary team with structured nutritional counselling can produce sustained, clinically meaningful improvements in dietary habits and biomarker profiles and are associated with better attainment of secondary prevention targets [16,18].

For clinical practice, this implies three actionable priorities:

(1) Promote a Mediterranean-style, whole-food dietary pattern as part of routine secondary prevention counselling;
(2) Embed dietitians and structured nutritional programmes within cardiac rehabilitation to support behaviour change and weight management;
(3) Individualise advice (including attention to comorbidity, cultural preferences and caloric goals) while monitoring adherence and risk factors over time [3,16,17,18].

For research, there remains a need for large, long-term pragmatic trials that compare realistic, implementable dietary interventions and that measure hard cardiovascular endpoints, cost-effectiveness, and strategies to improve sustained adherence across diverse populations. Until such data are available, combining guideline-recommended medical therapy with targeted, evidence-based dietary and lifestyle programmes offers the best current strategy to reduce recurrent events and improve long-term prognosis after ACS [1,3,7,12,13,16,17,18,19].

## Figures and Tables

**Table 1:** Overview of major dietary patterns, their key components, and associations with cardiovascular disease risk.

Name of Diet	Principle Components	Evidence for Use in Cardiovascular Disease
Mediterranean	This diet is characterised by a high intake of: [6] Plant foods (including fruits and vegetables); Breads and other cereals (traditionally minimally refined); Potatoes, beans, nuts, and seeds; Olive oil (especially virgin and extra-virgin olive oil) as the principal source of fat. A moderate intake of: Dairy products (mostly as cheese and yoghurt); Eggs (zero to four a week); Fish and poultry consumed in low to moderate amounts; Wine (particularly red, and consumed during meal times); Sweets containing sugars or honey a few times per week. A low intake of: Red meat. Typically, all the foods should be minimally processed, seasonally fresh, and locally grown.	Reduction in major cardiovascular events for both primary and secondary prevention [7,8,9]. Improves cardiovascular risk factors including cholesterol and lipid profile, blood pressure, glucose metabolism and insulin sensitivity [10,11,12].
Vegetarian	This refers to a diet in which all meats are excluded. Different subtypes include [13]: Ovolactovegetarian—excludes all meat from the diet, but allows for the consumption of products of animal origin, such as eggs and dairy products; Lactovegetarian—which excludes meat and eggs, but includes dairy products; Ovovegetarian—excludes meat and dairy products, but includes eggs; Pescovegetarian—individuals who exclude red meat and poultry from the diet but consume fish; Vegans—excludes all foods of animal origin; Flexitarian—allows for meat consumption sporadically or even multiple times per week.	Effective primary prevention against major cardiovascular events, particularly ischaemic heart disease [14,15]. Improves cardiovascular risk factors, including cholesterol and lipid profile, blood pressure, glucose metabolism, and weight loss [16,17].
Ketogenic	This diet is characterised by the rigorous limitation of carbohydrates while allowing a liberal ingestion of fats (including saturated fats) [18]. The aim is to place the body in a state of nutritional ketosis; to achieve this, the proportion of energy coming from fat in such a diet is usually 70–80%, with protein around 20%, and carbohydrates rarely exceeding 50 g per day [7,19].	The ketogenic diet has a beneficial effect on the improvement of cardiovascular risk factors, including blood pressure and weight loss [19]. Furthermore, it is associated with a reduction in serum Haemoglobin A1c (hence improved glycaemic control) and a substantial rise in high-density lipoprotein cholesterol levels [7].
Western	This is a dietary pattern characterised by high intakes of [8]: Pre-packaged foods; Refined grains; Meat—mostly red and processed as well as conventionally raised animal products, high-fat dairy products; High-sugar (and -fructose) food and drink; Fried foods.	No cardiovascular benefits, but rather, it has been associated with an increased cardiovascular risk, through the increased incidence of coronary artery disease [9,20] and a worse cardiovascular risk profile, including high blood pressure, adverse lipid profile, and an increased incidence of insulin resistance and type 2 diabetes [21,22].
Palaeolithic	This is a diet which is based on the eating patterns of humans in the Palaeolithic era, which encourages the intake of wild animal and plant foods [23]. Typically, this will include: Lean meats (especially wild game or grass-fed animals); Fish and seafood Fruits and vegetables Nuts and seeds Eggs Healthy fats (e.g., from avocados, olive oil, coconut oil). Foods excluded consist of: Grains (wheat, rice, oats, corn) Legumes (beans, lentils, peanuts, soy) Dairy products Refined sugar and salt Processed foods, oils, and additives Alcohol	Whilst there is limited data to support direct cardiovascular benefits of this diet, there is evidence to support an improvement of cardiovascular risk factors, including weight loss, blood pressure reduction, lower total cholesterol, and an improvement in glycaemic control [24,25,26,27].
Low-Fat	This consists of diets which limit total dietary fat to ≤ 30% of the total daily energy intake [28].	Improves cardiovascular risk factor of lipid profile [29]
(Very-) Low-Calorie	These diets are based on quantifying and consequently restricting the total daily calorie intake. Low-calorie: defined as up to 1200–1800 calories daily Very-low-calorie: defined as a total daily intake of <800 kcal per day [30]	Improves cardiovascular risk factors, including cholesterol and lipid profile, blood pressure, and weight loss, though these can be transient when there is subsequent weight gain upon withdrawal from the diet [31,32,33].
Personalised	No formal definition of this dietary type currently exists. Nonetheless, this diet encompasses the approach of using information on individual characteristics to develop targeted nutritional advice, products, or services [34].	This will vary according to the diet type.
Intermittent fasting	This eating regime commonly consists of a daily fast for 16 h, a 24 h fast on alternate days, or a fast two days per week on non-consecutive days [35].	Improves cardiovascular risk factors, including cholesterol and lipid profile, blood pressure, and weight loss [36,37].

## Data Availability

Data is contained within the article.
